# Statistically driven automated method for catalytic glucose conversion optimisation†

**DOI:** 10.1039/d4ra06038e

**Published:** 2024-11-07

**Authors:** Joseph Install, Rui Zhang, Jukka Hietala, Timo Repo

**Affiliations:** a Department of Chemistry, University of Helsinki A. I. Virtasen aukio 1, P.O. Box 55 00014 Finland timo.repo@helsinki.fi; b Neste Oyj, Technology Centre Kilpilahti, P.O. Box 310 06101 Porvoo Finland

## Abstract

A statistically driven, automated approach to optimize glucose transformations to platform chemicals, methyl lactate and levulinic acid, is reported. The combination of a robotic synthesis platform with design of experiments methods enabled efficient and precise modelling of glucose conversion catalysed by SnCl_4_·5H_2_O with 0–100% H_2_O and methanol as a cosolvent. Using this strategy, optimal reaction conditions within the available reaction space were identified in 58 runs, showcasing the excellent efficiency of this method in producing high yields of methyl lactate (75.9%) and levulinic acid (64.5%) in independent reactions *via* distinct retro-aldol condensation and dehydration pathways, respectively.

## Abstract 2

A statistically driven, automated approach to optimize glucose transformations to platform chemicals, methyl lactate and levulinic acid, is reported.

## Graphical abstract

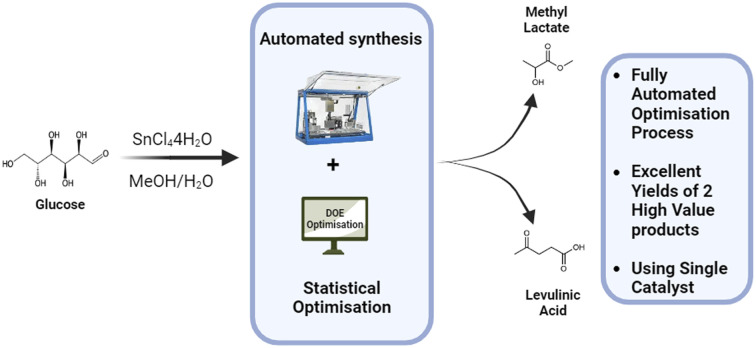

The pursuit of renewable and sustainable sources of platform chemicals is prevalent in modern chemistry. Accordingly, attention has been focused on nature to find alternatives to petrochemicals. Lignocellulosic biomass has long been a forerunner, given that it is composed of a plethora of structures.^1^ Lignin is a potential source for phenolic compounds,^2–4^ whereas cellulose and hemicellulose are sources of carbon fragments such as alcohols and carboxylic acids, formed through hydrolysis and fragmentation.^5^ These polysaccharides can be hydrolysed to yield single monosaccharide units, which have significant valorisation potential.^6,7^

One pursuit is retro-aldol condensation (RAC), utilising the carbonyl group to cleave a carbon–carbon bond in the sugar framework. This yields C2 (glyceraldehyde), C3 (glycolaldehyde) and C4 (aldotetrose) fragments in varying amounts, which through subsequent reactivity make up a considerable amount of a catalogue of sugar conversion products known today.^8,9^ Valuable C3 examples of RAC products are lactic acid and alkyl lactates, which are important building blocks for biodegradable polymers and in the cosmetic industry.^10^ In this case, alkyl lactates have distinct stability advantages compared to lactic acid.

A fundamental conversion for the evolution of high-value platform chemicals from carbohydrate sources is the dehydration (DeH) of C5 and C6 sugars to furanic ring structures. In the case of xylose C5 and glucose C6, these structures are commonly furfural and HMF, respectively.^8^ Following the rehydration of HMF, levulinic acid (levA) is formed. Similar to lactic acid, levA has significant industrial potential uses ranging from resins, plasticizers, and textiles to animal feed.^11^

Both RAC and DeH are widely studied in previous reports;^12–18^ however, their comparison as competing pathways, to the best of our knowledge, is underreported. In this work, we used a single Lewis acid catalyst, SnCl_4_·5H_2_O, which is active in both routes, and concluded through extensive screening that a close correlation exists between H_2_O concentration and product distribution.

We developed a new process (Fig. 1), which combines modern laboratory automation apparatus (Chemspeed Swing automated synthesis table) with a design of experiment (DoE) statistical experiment modelling software, to model the effect of the concentration of water in methanol on the SnCl_4_·5H_2_O-catalysed conversion of glucose into RAC (lactic acid and methyl lactate) and DeH (levA, methyl levulinate and HMF) with temperature as a co-parameter. As these are two of the major conversion routes for glucose, we believe that the developed process will provide insights into achieving higher efficiency processes for future chemical innovations. This closed-loop process provides unambiguous and clear representation of product yields, providing some predictability to these selected carbohydrate conversions.

**Fig. 1:**
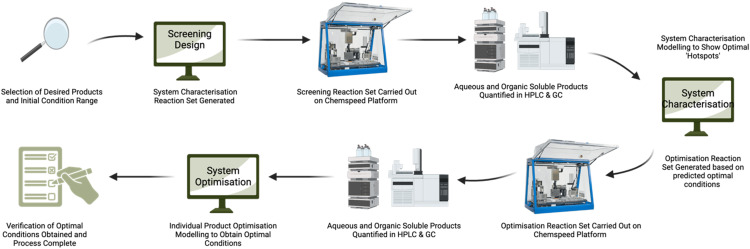
Combination of the design of experiments and automated synthesis process scheme used for the modelling of catalytic conversion of glucose.

To encompass the full reaction space, four full factorial experimental designs were used for the characterisation of glucose/SnCl_4_·5H_2_O with 0–100% water in methanol. The simultaneous quantification of lactic acid and methyl lactate by HPLC and GC was carried out, which are representative of the retro-aldol condensation products, and levulinic acid, methyl levulinate and HMF are representative of the dehydration products. This enabled the generation of a statistical model of the effects of water concentration and temperature for the full spectrum of values with just 30 reaction runs.

## Design of experiment statistical modelling

In this work, we utilised empirical modelling methods through the design of experiments (DOE) software package Modde Pro by Sartorius. The basic methodology of the modelling process is creating a statistical model set of the experimental region given by the user-inputted variables of the reaction and modelling the quantitative effects they have on the defined response.^19^ This operates in a more efficient and quantifiable manner than the one-factor at a time (OFAT) reaction optimisation traditionally used in the optimisation of chemical synthesis. DOE does this by creating a linear or quadratic correlation between the data input into the model.^20^ The reaction set that is generated is based on the selected model, for example, a full factorial model (Fig. SI1†). This would be a defined combination of the minimum and maximum of each parameter with centre points within the experimental region to enhance the correlation and provide a better estimation of the response in the full experimental region. The response in this work was the yields of the various identifiable products.

For the conversion of glucose into the products of retro-aldol condensation to C3 fragments and dehydration to furanic products, our chosen parameters or variables were the reaction temperature and the wt% of H_2_O with respect to methanol. The full system characterisation with one quadratic model would produce a low-quality model of the complete range of water percentage. Thus, to overcome this, we divided the wt% of H_2_O from 0–100 into 4 different full factorial models with reaction temperature as the other parameter (temperature of 140–180 °C). Consequently, we created a pseudo-polynomial function of the responses using four individual quadratic models. Each full factorial design requires 30 reactions with 3 centre points for accuracy. Given that the maximum values for blocks 1, 2, and 3 are the minimum for the next successive blocks, this reduced the total number of reaction runs required. This concept is described in Fig. SI2.†

Once the system characterisation was completed for all the observed products and the ‘hotspots’, which are areas of interest for individual products, were identified, subsequent optimisation reaction sets were generated to be carried out on the Chemspeed platform. These are described for methyl lactate and levA in more detail in the next section. For the optimisation DOE modelling, we selected a D-optimal design (Fig. SI3†). This was more suitable for the fine parameter optimisation after the near optimal parameter combination was obtained from the system characterisation.^21,22^ This allowed the synthesis to be optimised in a total of 58 reaction runs per target compound.

## Chemspeed automated reaction procedure

In this work, a Chemspeed Swing platform equipped with volumetric liquid handling and gravimetric solid dispensing systems as well as a Biotage initiator microwave reactor were used. The application was designed using the Chemspeed Autosuite Application Editor. Each reaction was run in succession from reaction mixture preparation, microwave reaction and subsequent dilution for analysis, which were all carried out by the automated platform. The run order was randomised by the Modde Pro DOE software. Although human bias is removed in automated synthesis, this was adhered to when preparing the system application for the Chemspeed platform (more detailed workflow described in ESI,† including system overview and application runs ESI 4 & 5†). Among the many benefits of using an automated synthesis platform, the ability to accurately dispense and vary the solvent conditions (ratio of H_2_O : MeOH) was highly beneficial for this procedure.

Once the reactions and sample preparation were completed, the aqueous samples were analysed using an Agilent 1200 series HPLC with DAD and RID detectors, equipped with a Thermo Scientific Acclaim mixed-mode WAX-1 HPLC column (120 Å, 4.6 × 150 mm) with a 5% ACN phosphate buffer solution at pH 5.5. DAD was used to detect the aqueous product components and quantify them by using an external calibration curve. Conversions were also calculated using HPLC for glucose quantification using an RID detector for each sample. In the case of the organic soluble products (methyl esters of organic acids and HMF), GC-FID was used with an external calibration curve and internal standard for quantification. Subsequently, the quantified data was used to generate the DOE models for characterisation and optimisation of the model reaction sets.

## Retro-aldol condensation

**Scheme 1:**
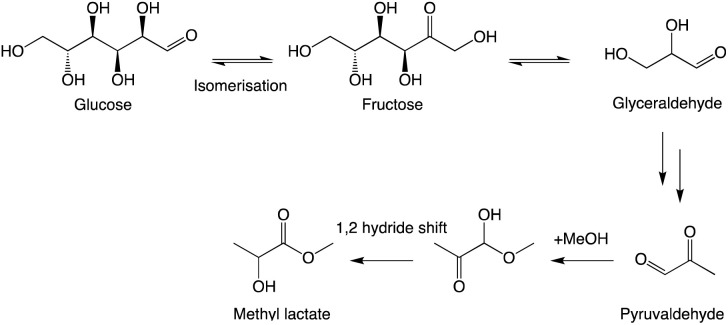
Methyl lactate formation mechanism from glucose.

After reviewing previous reports and preliminary screening of several metal chlorides, it was identified that SnCl_4_·5H_2_O was the most active in the RAC conversion of glucose.^14^ Through Brønsted acid-catalysed isomerisation of glucose to fructose, the subsequent cracking yields a C3 fragment, glyceraldehyde. After dehydration, pyruvaldehyde is formed and esterified in the presence of methanol and a 1,2-hydride shift yields methyl lactate (Scheme 1).^23^

The SnCl_4_·5H_2_O species are assumed to be dissociated in aqueous media and Sn^4+^ cations are shown computationally to effectively coordinate with the carbonyl groups of pyruvaldehyde^24^ and assist in the esterification of this species.

**Fig. 2:**
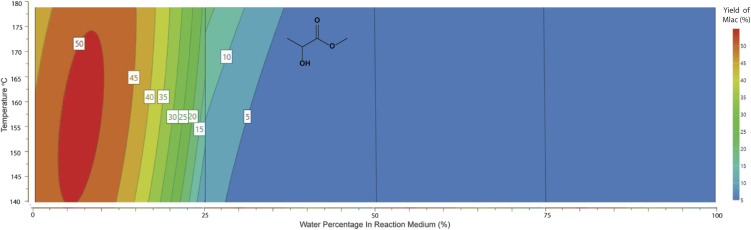
Response contour plot of methyl lactate yield (mol%) in response to water percentage (3 mL total volume) and temperature. Reaction conditions: glucose 0.5 mmol, SnCl_4_·5H_2_O 15 mol%, 3 mL reaction volume of MeOH/H_2_O, temperature 140–180 °C, and 15 min MW.

For the production of methyl lactate, methanol was the solvent of choice. However, preliminary studies showed that the addition of H_2_O in small amounts enhanced the conversion to methyl lactate. Our statistical design clarified this observation and allowed further investigation and optimisation. As shown in the contour plot in Fig. 2, a low water percentage is correlated with a higher proportion of RAC products, with the maximum methyl lactate yield predicted to be in the range of 45–50% with a water percentage of 2.5–10%. No lactic acid was identified in the high-water percentage area, with small amounts of *ca.* 5% detected in the range of 0–25% H_2_O (contour plot shown in ESI 7†). This is in contrast with publications with the sole scope of lactic acid production using tin chloride systems in pure water.^23,25^ A conclusion from the system characterisation is that small water concentrations aid the conversion to methyl lactate, which can be attributed to the dissolution of the sugar as well as the solvation of SnCl_4_. This observation is consistent with literature studies on the solvation of glucose and SnCl_4_ in methanol and water solutions.^26,27^

**Fig. 3:**
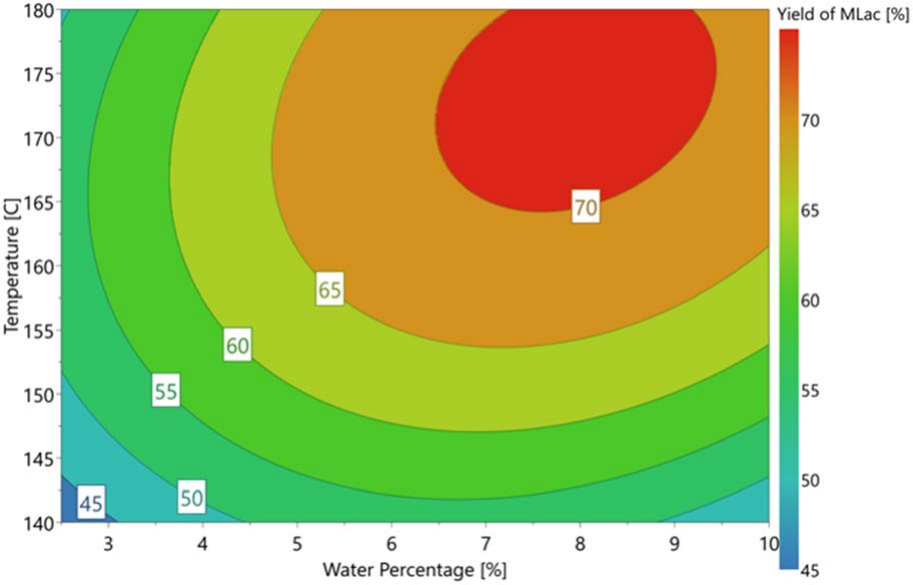
D-optimal response contour plot for optimal water percentage and temperature for methyl lactate production. Reaction conditions: glucose 0.5 mmol, SnCl_4_·5H_2_O 15 mol%, 3 mL reaction volume of MeOH/H_2_O, temperature 140–180 °C, and 15 min MW.

Based on this observation, an optimisation design of experiment model using the D-optimal model was produced and completed on the automated synthesis table, yielding the optimal response plot shown in Fig. 3. This was to find the optimal water percentage for the highest yield of methyl lactate. The maximum yield of 75.9% of methyl lactate was achieved with 7.5% H_2_O (3 mL total volume and methanol as the co-solvent). The reaction temperature was 180 °C.

Compared to the maximum yield under identical conditions in pure H_2_O, this enhancement to 75.9% yield with 7.5% water (2.775 mL : 0.225 mL MeOH : H_2_O) is significant and indicates the large effect of the addition of a small volume of water. The 7.5% water in the reaction mixture translates to 0.25 mL, which can be considered stochiometric with respect to the glucose starting material (100 mg). Although the addition of H_2_O is not required in the reaction pathway, it can be assumed necessary for the dissolution of glucose. A reaction at 5 times (500 mg glucose) the scale carried out in these experiments was carried out using the conventional heating method, which led to the yield of 58.8% methyl lactate.

## Dehydration and rehydration to levulinic acid

**Scheme 2:**
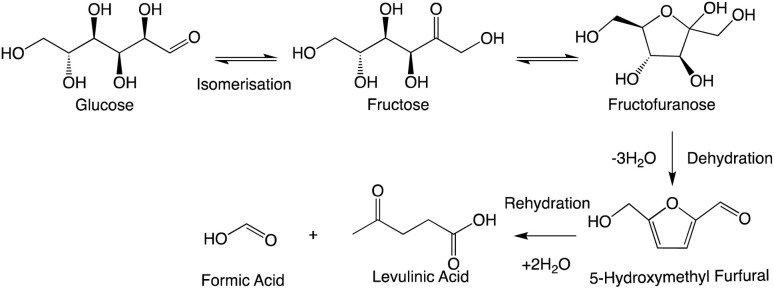
Levulinic and formic acid formation mechanism from glucose.

Through the dehydration pathway, it is also well agreed that an initial isomerisation reaction to fructose takes place, allowing ring-closing etherification to produce fructofuranose.^28^ This was shown *via* computational calculations to be the rate-determining step^29^ and to be lower in energy in the presence of Brønsted and/or Lewis acids,^30,31^ which SnCl_4_·5H_2_O will provide in aqueous media. The proceeding step in the dehydration introduces aromaticity in the form of HMF (contour plot shown in ESI 6†). HMF is stable enough for detection by HPLC and GC but unstable for extended periods. It is susceptible to a rehydration, a ring-opening pathway to produce levA and formic acid (Scheme 2).^32^

**Fig. 4:**
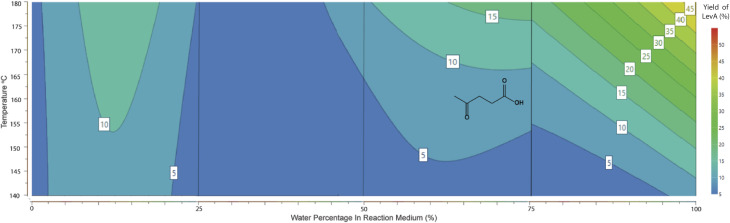
Response contour plot of levulinic acid yield (mol%) in response to water percentage (3 mL total volume) and temperature. Reaction conditions: glucose 0.5 mmol, SnCl_4_·5H_2_O 15 mol%, 3 mL reaction volume of MeOH/H_2_O, temperature 140–180 °C, and 15 min MW.

According to the contour plot indicating the effect of water concentration and temperature on levA yield (Fig. 4), it was apparent that the most suitable conditions are pure H_2_O and high temperature. The upper limit used was 180 °C, given that this was an acceptable limit for vapour pressure in the presence of methanol in other parts of the experimental region. An observation from the reaction products was the formation of an insoluble precipitate. This was suspected to be an insoluble Sn complex. X-ray diffraction and FT-IR were used to confirm that the identity of the precipitate was SnO_2_ (shown in ESI 11†). Tin oxide is insoluble in water and methanol, and therefore its formation is considered the deactivation of the Sn catalyst and its ability to effectively coordinate with the reaction intermediates.

To reduce the formation of precipitates and retain the catalytic activity, HCl was used as an additive to maximise the yield of levA.^12^ A D-optimal DoE model was produced and carried out with HCl addition corresponding to the molar ratio with respect to the catalyst in the range of 1 : 5 (40 μL) to 1 : 15 (120 μL) and temperature (temperature range of 180–220 °C). The effect of these two factors on the yield was measured and used to obtain the optimal conditions.

**Fig. 5:**
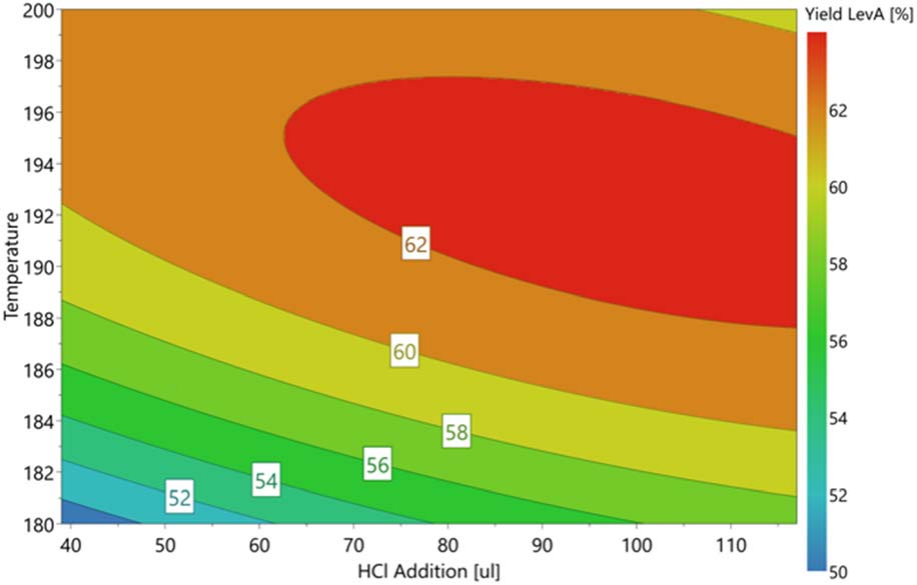
D-optimal response contour plot for optimal HCl addition and temperature for levulinic acid production. Reaction conditions: glucose 0.5 mmol, SnCl_4_·5H_2_O 15 mol%, 3 mL reaction volume of H_2_O, temperature 180–200 °C, and 15 min MW.

The results of the D-optimal plot for levA (Fig. 5) show that a high molar ratio of HCl increases the Brønsted acidity of the system to allow extended activity of the catalyst. The reaction of SnO_2_ with HCl regenerates SnCl_4_ according to the equilibrium.SnO_2_·*x*H_2_O + 4HCl ⇌ SnCl_4_ + (*x* + 2)H_2_O

Utilising this, we could achieve the maximum yield of 63.5% levA with the addition of 65 μL HCl, which is a significant increase compared to identical conditions without HCl (50.5% with no HCl added). These results also emphasise the ability of aqueous Sn species to catalyse the conversion of glucose.

## Comparison of DeH and RAC pathway mol efficiency

**Fig. 6:**
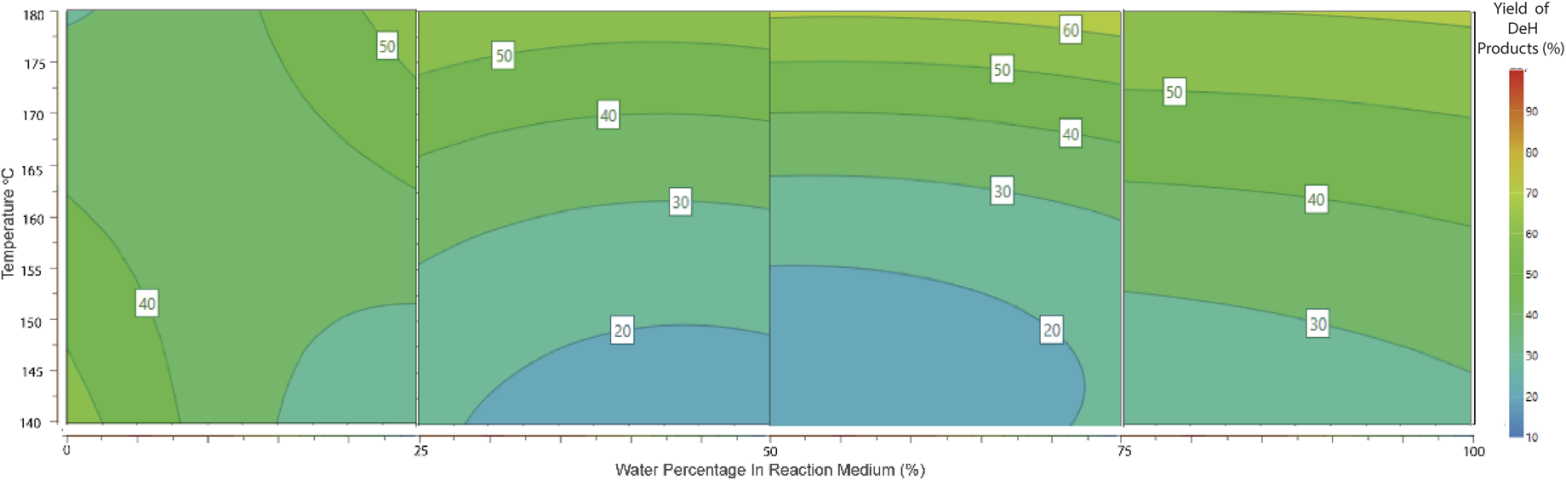
Response contour plot of combined dehydration product (HMF, MMF, and levulinic acid) yield (mol%) in response to water percentage (3 mL total volume) and temperature. Reaction conditions: glucose 0.5 mmol, SnCl_4_·5H_2_O 15 mol%, 3 mL reaction volume of MeOH/H_2_O, temperature 140–180 °C, and 15 min MW.

**Fig. 7:**
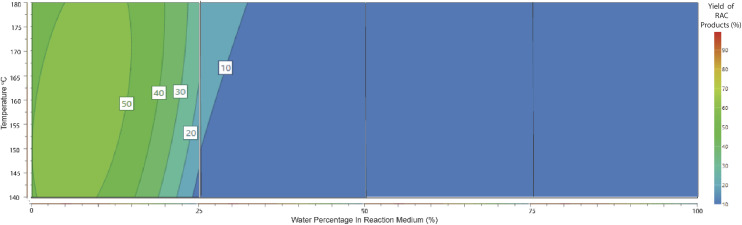
Response contour plot of combined retro-aldol condensation products (methyl lactate and lactic acid) yields (mol%) in response to water percentage (3 mL total volume) and temperature. Reaction conditions: glucose 0.5 mmol, SnCl_4_·5H_2_O 15 mol%, 3 mL reaction volume MeOH/H_2_O, temperature 140–180 °C, and 15 min MW.

During the modelling procedure, the molar yields of 5 products were recorded simultaneously and fit to the quadratic model in each of the 4 full factorial blocks. This allowed a comparison of the efficiency of each reaction pathway across a range of water percentages. The utilisation of 4 separate quadratic models allowed a pseudo-polynomial representation of this reactive space. A visual representation is presented in Fig. 6 and 7. In the case of RAC conversion, it is evident that a low water percentage is favourable, and the yields dissipate as the water concentration increases, suggesting that the presence of water interferes with the C–C bond cleavage ability or the pathway is outcompeted by the dehydration pathway. Regarding the dehydration pathways, product evolution can be seen across the full range, foremost suggesting that this is the most efficient product pathway. Also, the competing effect of the RAC pathways on dehydration is seen, where the area of high RAC product evolution is matched with reduced DeH in the respective block.

## Conclusions

Our novel statistical automated method allowed an efficient system characterisation across a full range of water concentrations. As a result, outstanding yields in SnCl_4_·5H_2_O-catalysed glucose conversions in H_2_O/MeOH were identified. The yield of 75.9% methyl lactate was achieved by finely tuning the MeOH to water ratio to a value of 7.5% volume of H_2_O with methanol in a 92.5% solvent composition. A comparison with previous work^14,33^ is presented in Table 1. In the case of levulinic acid, we achieved a yield of 63.5% independently *via* the addition of HCl to 100% H_2_O reaction medium. This is comparable to similar works in alternative media using SnCl_4_ as the catalyst^18^ and similar work in Table 1. The addition of HCl proved beneficial for the catalytic reaction, with the optimal loading being approximately 9 molar equivalents with respect to the catalyst. Presumably, the addition of HCl improves the stability of the catalyst by reducing the formation of tin oxide species under aqueous conditions.

**Table 1:** Comparison of similar systems using homogenous catalysts for the production of methyl lactate and levulinic acid

Product	Catalyst	Solvent	Yield	Ref.
Methyl lactate	InCl_2_	MeOH	36%	14
Ethyl lactate	ZnCl_2_	EtOH (3% H_2_O)	48%	34
Methyl lactate	SnCl_4_·5H_2_O	MeOH (7.5% H_2_O)	76%	This work
Levulinic acid	CrCl_3_, HCl	H_2_O/THF	46%	35
Levulinic acid	H_2_SO_4_	H_2_O	38%	36
Levulinic acid	SnCl_4_·5H_2_O	H2O	64%	This work

Enabled by statistical modelling, each optimum was obtained in 14 reaction runs following the initial 30 runs for the system characterisation. Modern automation techniques enable significant savings in terms of time with minimal human interaction. We believe that these methods using a combination of DoE modelling and automation as an optimisation tool can help accelerate process optimisation and selectivity control, thus speeding up the development of a more sustainable chemical industry.

## Data availability

The data used for the preparation of this communication are listed in the ESI section.† The software used is a commercial package from Sartorius Modde Pro and AutoSuite from Chemspeed technologies.

## Author contributions

Joseph Install: conceptualization, investigation, methodology, data curation, formal analysis, software, writing – original draft, writing – review & editing; Dr Rui Zhang: investigation, methodology, data curation, formal analysis, software, writing – review & editing; Dr Jukka Hietala: conceptualization, writing – review & editing; Prof. Timo Repo*: conceptualization, funding acquisition, supervision, resources, project administration, writing – review & editing.

## Conflicts of interest

There are no conflicts to declare.

## Supplementary Material

RA-014-D4RA06038E-s001
