# Supplementary material for: Statistically driven automated method for catalytic glucose conversion optimisation
Source: RSC Adv. 2024 Nov 7;14(48):35578–84. doi: 10.1039/d4ra06038e (PMC11542708; doi:10.1039/d4ra06038e)
Supplement: RA-014-D4RA06038E-s001 [file RA-014-D4RA06038E-s001.pdf]

Supplementary Information (SI) for RSC Advances.
This journal is © The Royal Society of Chemistry 2024

Supporting Information

Statistical driven automated method for catalytic glucose conversion optimisation

Joseph Install[a,1], Rui Zhang[a,1], Jukka Hietala[b] and Timo Repo[a]*

a) Department of Chemistry, University of Helsinki, A. I. Virtasen aukio 1, P.O. Box 55, 00014 Helsinki (Finland), E-mail: timo.repo@helsinki.fi.

b) Neste Oyj, Technology Centre, Kilpilahti, P.O. Box 310, 06101 Porvoo, Finland

1) Authors J.Install and R.Zhang Contributed Equally

*Corresponding author: timo.repo@helsinki.fi

1. Design of experiment methods

1.1 Full Factorial Design

All statistical models were generated using Umetrics Sartorius Modde Pro software. The system characterisation resulting in figures 2, 4, 6 and 7 were using the full factorial model shown in SI1. The method allows efficient screening of multiple optimisation parameters simultaneously in the experimental region by creating a quadratic model using the high and low values of each parameter in different permutations as well as middle point to increase the accuracy of the model. This then allows a response to be predicted within the region, the response within this work is the respective yield of methyl lactate, lactic acid, HMF, levulinic acid and methyl levulinate.

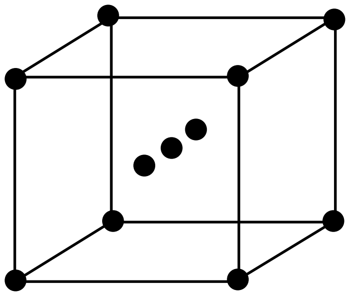

SI 1: *Full Factorial statistical design of the experimental region*

1.2 Combination of multiple full factorial design

Within the study the aim was to model the effect of water concentration on the product distribution using a single Lewis acid catalyst for glucose conversions. Using a single full factorial design and considering the highest power model is quadratic, this would likely return a 'false' peak or lack resolution. Therefore, placing four sequential full factorial designs together as outlined in SI2, allowed a pseudo polynomial model to be created. By combining these models, it also boosted reliability as the corners of each design cube lay within the model space for the next and thus was a repeat for each of these data points. The surface plots of the individual full factorial designs were then placed in order and the full water concentration spectrum was created.

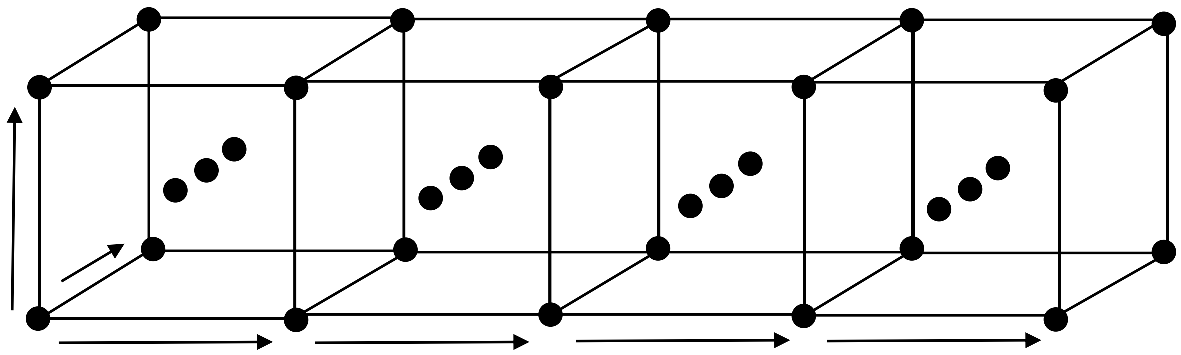

SI 2: *Combination of 4 Full Factorial statistical design of the experimental region*

1.3 D-Optimal Design for Optimised Results

From the outputs of the system characterisation, it was then possible to identify areas of high yields for each respective product. To gain a true optimal prediction a different design of experiment model is required; D-optimal design. This is outlined in SI3 and the points are generated by the statistical software with a model designed to find the optimum.

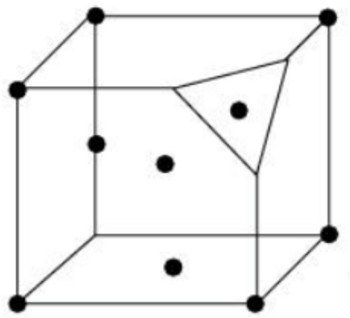

SI 3: *D-optimal statistical design of the experimental region*

2. Experimental Trials

2.1 Materials

Analytical standards of Levulinic acid, methyl lactate and lactic acid were purchased from Tokyo Chemical Industry Europe (TCI), methyl levulinate, HMF were purchased from Sigma Aldrich. Glucose and $SnCl_4 \cdot 6H_2O$ were purchased from Sigma Aldrich. Methanol and ethyl acetate (HPLC grade) were purchased from Fisher Scientific, H_2O used in reactions and HPLC analysis was purified MilliQ type 1.

2.2 Automated Chemspeed Swing Synthesis Platform

Experimental runs generated by the statistical modelling software when transferred into an application to run of a Chemspeed Automated Swing platform. The platform is equipped with volumetric liquid handling and gravimetric solid dispensing systems as well as a biotage initiator microwave reactor. Each reaction run was carried out independently under identical controlled conditions. The platform also has workup abilities which added to the automation level of this process.

An example reaction is displayed in the schemes SIX and SIY from the AutoSuite editor on the chemspeed software. Using the gravimetric dispensing unit GDU, the solid materials, glucose and $SnCl_4 \cdot 6H_2O$ were dispensed in the predefined amounts (100mg and 30mg respectively), into a microwave vial equipped with stirrer bar. After, using the 4-needle head (4NH) coupled with syringe dispensing system, the solvent mixture is dispensed in the amounts defined in the statiscal reaction set, for example 2.25mL MeOH and 0.75mL H_2O to make the total reaction volume 3mL. Once the reaction mixture is prepared, using the crimp and transport tool, the vial is capped and transferred to the microwave reactor where the microwave reaction profile is controlled and defined in the AutoSuite editor. Once completed the vial is transferred back to the vial rack where, using the 4NH, 2 sequential analytical sample aliquots are collected through the septa in the microwave vial cap. In a separate 2 tier rack, the sample is diluted in respective GC and HPLC solvents (EtOAc & Phosphate Buffer 50mmol solution) and an internal standard is added to the

GC sample solution. Using the 4NH 1mL of each analytical sample is transferred to an analytical vial in the lower level of the 2-tier rack. These samples are now ready for injection into the GC and HPLC instruments for quantification of products. For efficiency purposes the analytical workup was done in batches of 5 completed reactions.

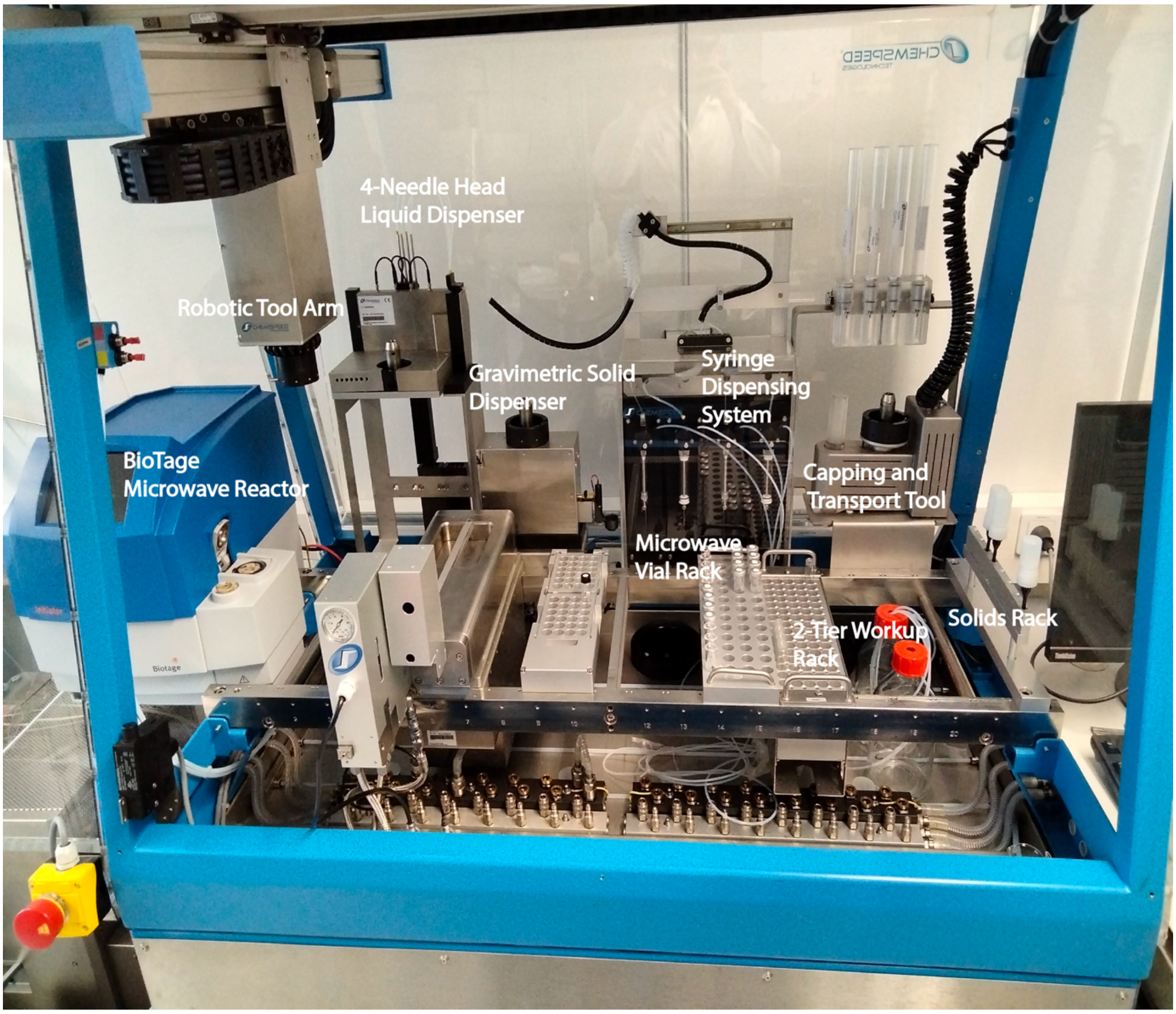

SI 4: *Chemspeed Swing Automated platform equipped with solid and liquid dispensers, microwave reactor*

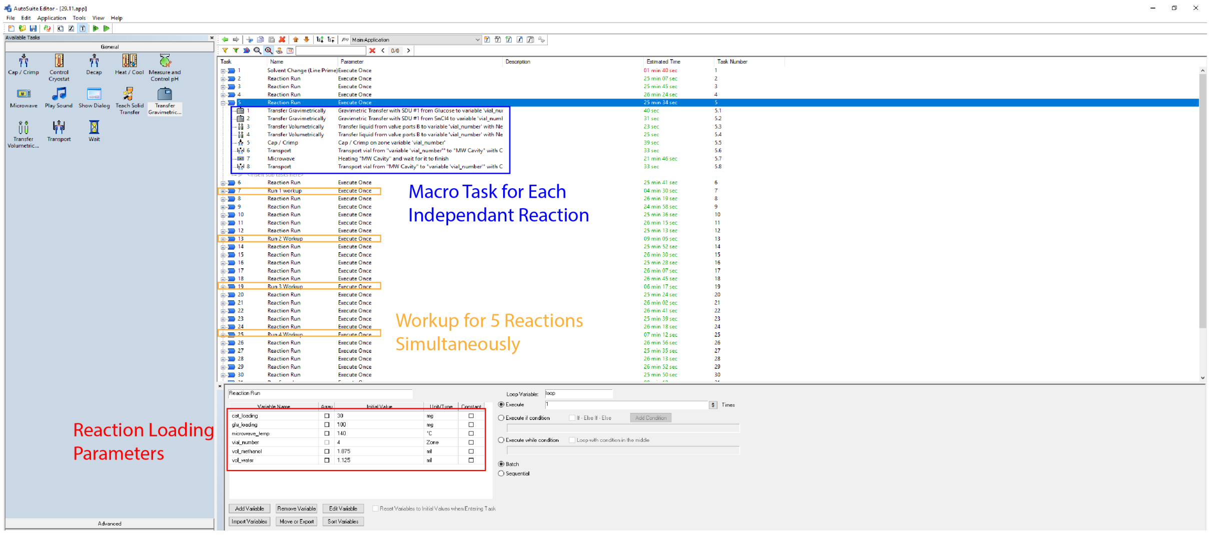

SI 5: Chemspeed Swing Automated platform application editor example for running optimisation reactions

***Table SI 1:** Quantities and volumes dispensed by the Chemspeed Automated Synthesis platform for the reaction optimization block*

Reaction ID	Glucose Loading / mg	Catalyst Loading / mg	Time MW (min)	Temperature C	MeOH %	Water %	Volume MeOH Dispensed / mL	Volume H2O Dispensed / mL	Glucose conversion %	Analytical Sample Total Volume GC / mL	Analytical Sample Total Volume HPLC / mL	Reaction Aliquot Volume GC / mL	Internal Standard (2,3,4-trichlorobenzene) Addition GC / mL	Reaction Aliquot Volume HPLC / mL
MB_1	100	30	15	140	100	0	3	0	100.000	5.0	5.0	0.3	0.1	0.5
MB_2	100	30	15	160	100	0	3	0	90.284	5.0	5.0	0.3	0.1	0.5
MB_3	100	30	15	180	100	0	3	0	78.839	5.0	5.0	0.3	0.1	0.5
MB_4	100	30	15	140	87.5	12.5	2.625	0.375	85.636	5.0	5.0	0.3	0.1	0.5
MB_5	100	30	15	160	87.5	12.5	2.625	0.375	81.495	5.0	5.0	0.3	0.1	0.5
MB_6	100	30	15	180	87.5	12.5	2.625	0.375	97.950	5.0	5.0	0.3	0.1	0.5
MB_7	100	30	15	140	75	25	2.25	0.75	60.070	5.0	5.0	0.3	0.1	0.5
MB_8	100	30	15	160	75	25	2.25	0.75	65.963	5.0	5.0	0.3	0.1	0.5
MB_9	100	30	15	180	75	25	2.25	0.75	85.107	5.0	5.0	0.3	0.1	0.5
MB_10	100	30	15	140	87.5	12.5	2.625	0.375	85.425	5.0	5.0	0.3	0.1	0.5
MB_11	100	30	15	160	87.5	12.5	2.625	0.375	95.469	5.0	5.0	0.3	0.1	0.5
MB_12	100	30	15	180	87.5	12.5	2.625	0.375	98.755	5.0	5.0	0.3	0.1	0.5
MB_13	100	30	15	140	62.5	37.5	1.875	1.125	26.967	5.0	5.0	0.3	0.1	0.5
MB_14	100	30	15	160	62.5	37.5	1.875	1.125	51.482	5.0	5.0	0.3	0.1	0.5
MB_15	100	30	15	180	62.5	37.5	1.875	1.125	79.330	5.0	5.0	0.3	0.1	0.5
MB_16	100	30	15	140	50	50	1.5	1.5	19.713	5.0	5.0	0.3	0.1	0.5
MB_17	100	30	15	160	50	50	1.5	1.5	40.218	5.0	5.0	0.3	0.1	0.5
MB_18	100	30	15	180	50	50	1.5	1.5	72.361	5.0	5.0	0.3	0.1	0.5
MB_19	100	30	15	140	37.5	62.5	1.125	1.875	28.753	5.0	5.0	0.3	0.1	0.5
MB_20	100	30	15	160	37.5	62.5	1.125	1.875	46.094	5.0	5.0	0.3	0.1	0.5
MB_21	100	30	15	180	37.5	62.5	1.125	1.875	75.850	5.0	5.0	0.3	0.1	0.5
MB_22	100	30	15	140	25	75	0.75	2.25	16.523	5.0	5.0	0.3	0.1	0.5
MB_23	100	30	15	160	25	75	0.75	2.25	47.104	5.0	5.0	0.3	0.1	0.5
MB_24	100	30	15	180	25	75	0.75	2.25	76.369	5.0	5.0	0.3	0.1	0.5
MB_25	100	30	15	140	12.5	87.5	0.375	2.625	20.031	5.0	5.0	0.3	0.1	0.5
MB_26	100	30	15	160	12.5	87.5	0.375	2.625	46.684	5.0	5.0	0.3	0.1	0.5
MB_27	100	30	15	180	12.5	87.5	0.375	2.625	76.952	5.0	5.0	0.3	0.1	0.5
MB_28	100	30	15	140	0	100	0	3	18.846	5.0	5.0	0.3	0.1	0.5
MB_29	100	30	15	160	0	100	0	3	46.183	5.0	5.0	0.3	0.1	0.5
MB_30	100	30	15	180	0	100	0	3	67.200	5.0	5.0	0.3	0.1	0.5

Solid Dispensed By Chemspeed Gravemetric Dispensing Unit

Liquid Dispensed by Chemspeed Volumetric Liquid Dispenser

Table SI 2: *Quantities and volumes dispensed by the Chemspeed Automated Synthesis platform for the methyl lactate optimization block*

Reaction ID	Glucose Loading / mg	Catalyst Loading / mg	Time MW (min)	Temperature C	MeOH %	Water %	Volume MeOH Dispensed / mL	Volume H2O Dispensed / mL	Glucose conversion %	Analytical Sample Total Volume GC / mL	Analytical Sample Total Volume HPLC / mL	Reaction Aliquot Volume GC / mL	Internal Standard (2,3,4-trichlorobenzene) Addition GC / mL	Reaction Aliquot Volume HPLC / mL	Methyl Lactate Yield / mol %
MLO_1	100	30	15	140.0	97.5	2.5	2.925	0.075	93.7	5.0	5.0	0.3	0.1	0.5	37.5
MLO_2	100	30	15	140.0	90	10	2.7	0.3	94.0	5.0	5.0	0.3	0.1	0.5	42.8
MLO_3	100	30	15	180.0	97.5	2.5	2.925	0.075	98.4	5.0	5.0	0.3	0.1	0.5	46.9
MLO_4	100	30	15	180.0	90	10	2.7	0.3	98.0	5.0	5.0	0.3	0.1	0.5	68.6
MLO_5	100	30	15	166.7	97.5	2.5	2.925	0.075	96.2	5.0	5.0	0.3	0.1	0.5	56.6
MLO_6	100	30	15	153.3	90	10	2.7	0.3	95.7	5.0	5.0	0.3	0.1	0.5	58.8
MLO_7	100	30	15	166.7	90	10	2.7	0.3	96.3	5.0	5.0	0.3	0.1	0.5	59.6
MLO_8	100	30	15	140.0	92.5	7.5	2.775	0.225	94.4	5.0	5.0	0.3	0.1	0.5	51.4
MLO_9	100	30	15	180.0	95	5	2.85	0.15	98.5	5.0	5.0	0.3	0.1	0.5	57.8
MLO_10	100	30	15	180.0	92.5	7.5	2.775	0.225	98.2	5.0	5.0	0.3	0.1	0.5	74.5
MLO_11	100	30	15	160.0	93.75	6.25	2.8125	0.1875	95.9	5.0	5.0	0.3	0.1	0.5	64.4
MLO_12	100	30	15	160.0	93.75	6.25	2.8125	0.1875	95.6	5.0	5.0	0.3	0.1	0.5	64.2
MLO_13	100	30	15	160.0	93.75	6.25	2.8125	0.1875	96.5	5.0	5.0	0.3	0.1	0.5	64.0

Solid Dispensed by Chemspeed Gravemetric Dispensing Unit

Liquid Dispensed by Chemspeed Volumetric Liquid Dispenser

Table SI 3: *Quantities and volumes dispensed by the Chemspeed Automated Synthesis platform for the levulinic acid optimization block*

Reaction ID	Glucose Loading / mg	Catalyst Loading / mg	Time MW (min)	Temperature C	MeOH %	Water %	HCl Addition / mL	Volume MeOH Dispensed / mL	Volume H2O Dispensed / mL	Glucose conversion %	Analytical Sample Total Volume GC / mL	Analytical Sample Total Volume HPLC / mL	Reaction Aliquot Volume GC / mL	Internal Standard (2,3,4-trichlorobenzene) Addition GC / mL	Reaction Aliquot Volume HPLC / mL	Levulinic Acid Yield / mol %
LEVO_1	100	30	15	180.0	0	100	0.039	0	3	98.2	5.0	5.0	0.3	0.1	0.5	48.3
LEVO_2	100	30	15	200.0	0	100	0.039	0	3	100.0	5.0	5.0	0.3	0.1	0.5	57.3
LEVO_3	100	30	15	180.0	0	100	0.117	0	3	98.4	5.0	5.0	0.3	0.1	0.5	60.9
LEVO_4	100	30	15	200.0	0	100	0.117	0	3	99.4	5.0	5.0	0.3	0.1	0.5	60.8
LEVO_5	100	30	15	180.0	0	100	0.091	0	3	100.0	5.0	5.0	0.3	0.1	0.5	49.1
LEVO_6	100	30	15	200.0	0	100	0.065	0	3	100.0	5.0	5.0	0.3	0.1	0.5	63.0
LEVO_7	100	30	15	200.0	0	100	0.091	0	3	100.0	5.0	5.0	0.3	0.1	0.5	60.8
LEVO_8	100	30	15	186.6	0	100	0.039	0	3	93.5	5.0	5.0	0.3	0.1	0.5	53.2
LEVO_9	100	30	15	193.3	0	100	0.039	0	3	97.3	5.0	5.0	0.3	0.1	0.5	59.5
LEVO_10	100	30	15	193.3	0	100	0.117	0	3	100.0	5.0	5.0	0.3	0.1	0.5	57.2
LEVO_11	100	30	15	190.0	0	100	0.078	0	3	98.6	5.0	5.0	0.3	0.1	0.5	60.4
LEVO_12	100	30	15	190.0	0	100	0.078	0	3	98.6	5.0	5.0	0.3	0.1	0.5	62.0
LEVO_13	100	30	15	190.0	0	100	0.078	0	3	100.0	5.0	5.0	0.3	0.1	0.5	63.6

Solid Dispensed by Chemspeed Gravemetric Dispensing Unit

Liquid Dispensed by Chemspeed Volumetric Liquid Dispenser

***Table SI 4:** Mass Balance for the Main Block of Optimization carried out on the Chemspeed Platform*

Reaction ID	Glucose Loading / mg	Temperature C	Water / %	Conversion of Glucose / %	Mass Glucose Converted / mg	Mass Lactic Acid / Mg	Mass Levulinic Acid / mg	Mass HMF / mg	Mass Methyl Levulinate / mg	Mass Methyl Lactate / mg	Mass Formic / mg	Mass of Unidentified Products / Mg
MB_1	100	140	0	100.0	100.0	0.7	1.3	14.2	14.1	20.6	5.3	43.70
MB_2	100	160	0	90.3	90.3	1.4	2.6	9.2	20.8	28.6	8.0	19.57
MB_3	100	180	0	78.8	78.8	0.6	1.3	14.6	3.6	20.9	1.6	36.27
MB_4	100	140	12.5	85.6	85.6	2.3	6.3	12.1	5.7	25.7	3.5	30.00
MB_5	100	160	12.5	81.5	81.5	2.1	6.2	13.8	5.1	23.0	3.3	28.05
MB_6	100	180	12.5	97.9	97.9	3.0	9.2	8.7	7.0	30.2	4.7	35.12
MB_7	100	140	25	60.1	60.1	0.4	0.3	12.4	2.3	3.8	0.9	39.87
MB_8	100	160	25	66.0	66.0	1.6	2.5	13.0	8.1	5.6	3.4	31.81
MB_9	100	180	25	85.1	85.1	1.6	1.9	20.0	20.5	10.5	7.7	22.93
MB_10	100	160	12.5	85.4	85.4	1.7	4.7	11.4	5.6	27.0	3.1	31.90
MB_11	100	160	12.5	95.5	95.5	2.1	5.7	8.3	8.6	26.3	4.4	40.08
MB_12	100	160	12.5	98.8	98.8	3.1	8.7	8.5	10.4	31.8	5.8	30.53
MB_13	100	140	37.5	27.0	27.0	0.0	0.7	10.4	1.2	1.7	0.6	12.33
MB_14	100	160	37.5	51.5	51.5	0.0	1.6	14.1	4.6	1.2	2.0	27.94
MB_15	100	180	37.5	79.3	79.3	0.0	2.9	19.4	15.6	1.0	6.2	34.24
MB_16	100	140	50	19.7	19.7	0.0	1.0	10.0	0.0	2.4	0.2	6.05
MB_17	100	160	50	40.2	40.2	0.0	2.9	14.0	2.5	1.1	1.6	18.13
MB_18	100	180	50	72.4	72.4	0.0	4.1	19.7	16.2	0.7	6.7	24.85
MB_19	100	140	62.5	28.8	28.8	0.0	3.6	6.4	0.0	1.4	0.9	16.55
MB_20	100	160	62.5	46.1	46.1	0.0	2.6	12.2	1.8	1.1	1.3	27.16
MB_21	100	180	62.5	75.9	75.9	0.0	9.3	17.4	21.8	0.6	10.0	16.85
MB_22	100	140	75	16.5	16.5	0.0	1.2	10.7	1.7	0.8	0.9	1.17
MB_23	100	160	75	47.1	47.1	0.0	4.2	13.1	2.2	0.9	1.8	24.99
MB_24	100	180	75	76.4	76.4	0.0	11.6	13.8	18.7	0.6	9.4	22.24
MB_25	100	140	87.5	20.0	20.0	0.0	1.0	11.3	1.4	2.4	0.7	3.20
MB_26	100	160	87.5	46.7	46.7	0.0	8.4	14.3	2.6	0.9	2.9	17.59
MB_27	100	180	87.5	77.0	77.0	0.0	18.0	9.1	12.0	1.1	8.6	28.14
MB_28	100	140	100	18.8	18.8	0.0	5.2	13.6	0.0	0.0	1.3	0.00
MB_29	100	160	100	46.2	46.2	0.0	14.0	13.4	0.0	0.0	3.4	15.31
MB_30	100	180	100	67.2	67.2	0.0	41.5	7.6	0.0	0.0	10.0	8.08

***Table SI 5:** Mass Balance for the Optimization of Methyl Lactate carried out on the Chemspeed Platform*

Time (min)	Glucose Loading / mg (mg)	Catalyst Loading / mg (mg)	Temp / °C	Water %	Glucose Conversion / %	Mass HMF / mg	Mass LevA / mg	Mass Formic Acid / mg	Mass Mlev / mg	Mass Lactic Acid / mg	Mass of Mlac / mg	Mass of H_2O / mg	Total Mass / mg	Mass Unidentified Products / mg
5	100	30	180	7.5	100%	3.1	3.4	2.8	4.1	3.8	28.3	7.9	53.50	46.50
10	100	30	180	7.5	100%	3.1	5.4	7.0	13.8	5.1	35.5	11.0	80.92	19.08
20	100	30	180	7.5	100%	3.1	4.6	6.3	12.7	5.3	34.0	10.4	76.37	23.63
30	100	30	180	7.5	100%	2.7	4.1	6.4	13.4	5.0	34.0	10.2	75.70	24.30
5	100	10	180	7.5	100%	3.1	3.1	2.0	2.3	4.4	32.6	8.2	55.74	44.26
10	100	10	180	7.5	100%	2.9	2.9	4.4	9.1	2.9	24.7	7.7	54.44	45.56
20	100	10	180	7.5	100%	2.6	2.6	2.1	2.9	2.2	32.5	7.9	52.86	47.14
30	100	10	180	7.5	100%	2.8	2.8	2.1	2.8	1.2	32.5	8.1	52.27	47.73

2.3 Microwave Reactions

Microwave reactions were carried out in the biotage initiator microwave reactor controlled on the Chemspeed platform. These were fixed to 150W power and limited to 20 bar of pressure for saftey reasons.

3 Analysis and Quantifications

3.1 HPLC Analysis

For the organic acids HPLC analysis was carried out using an Agilent 1200 series HPLC with DAD and RID detectors equipped with a Thermo Scientific Acclaim mixed mode WAX-1 HPLC column (120am 4.6x150mm) with a 5% ACN phosphate buffer solution at pH 5.5. DAD was used to detect the product components and quantify using an external calibration curve. Conversions were also calculated using HPLC for glucose quantification using RID detector is each of the reaction samples.

Samples were prepared for analysis by extracting a 0.1mL aliquot and diluting to 5mL using the Chemspeed platform. This was then filtered using 0.2µm hydrophilic syringe filters and injected into the HPLC.

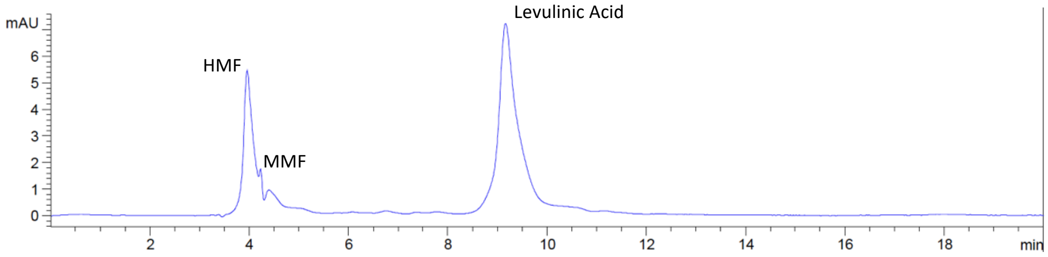

SI 6: *Example HPLC Chromatogram of the reaction mixture using Acclaim mixed mode WAX-1 column with a 5% ACN Phosphate Buffer Mobile Phase pH 5.5, UV 230nm. Reaction Conditions: 100mg glucose, 30mg $SnCl_4{\cdot}6H_2O$ with 3mL H_2O, 10min MW 180°C*

3.3 GC Analysis

The esters, methyl lactate, methyl levulinate and HMF were quantified using gas chromatography for peak definition and accuracy of quantification. For this an Agilent 7890B GC equipped with flame ionisation detector was used for high quality detection and quantification. The column used was an Agilent J&W GC HP Column, length 30m, diameter 0.25mm and 0.25um film. An internal standard of 2,3,5-trichloro benzene was used and quantifications made using external quantification with respect to the internal standard for high precision quantifications. The quantifications were also made in parallel with HPLC conversion analysis using the RID detection.

Examples GC-MS Chromatogram for the product identification, whereby quantifications carried out in GC-FID under identical column and methods.

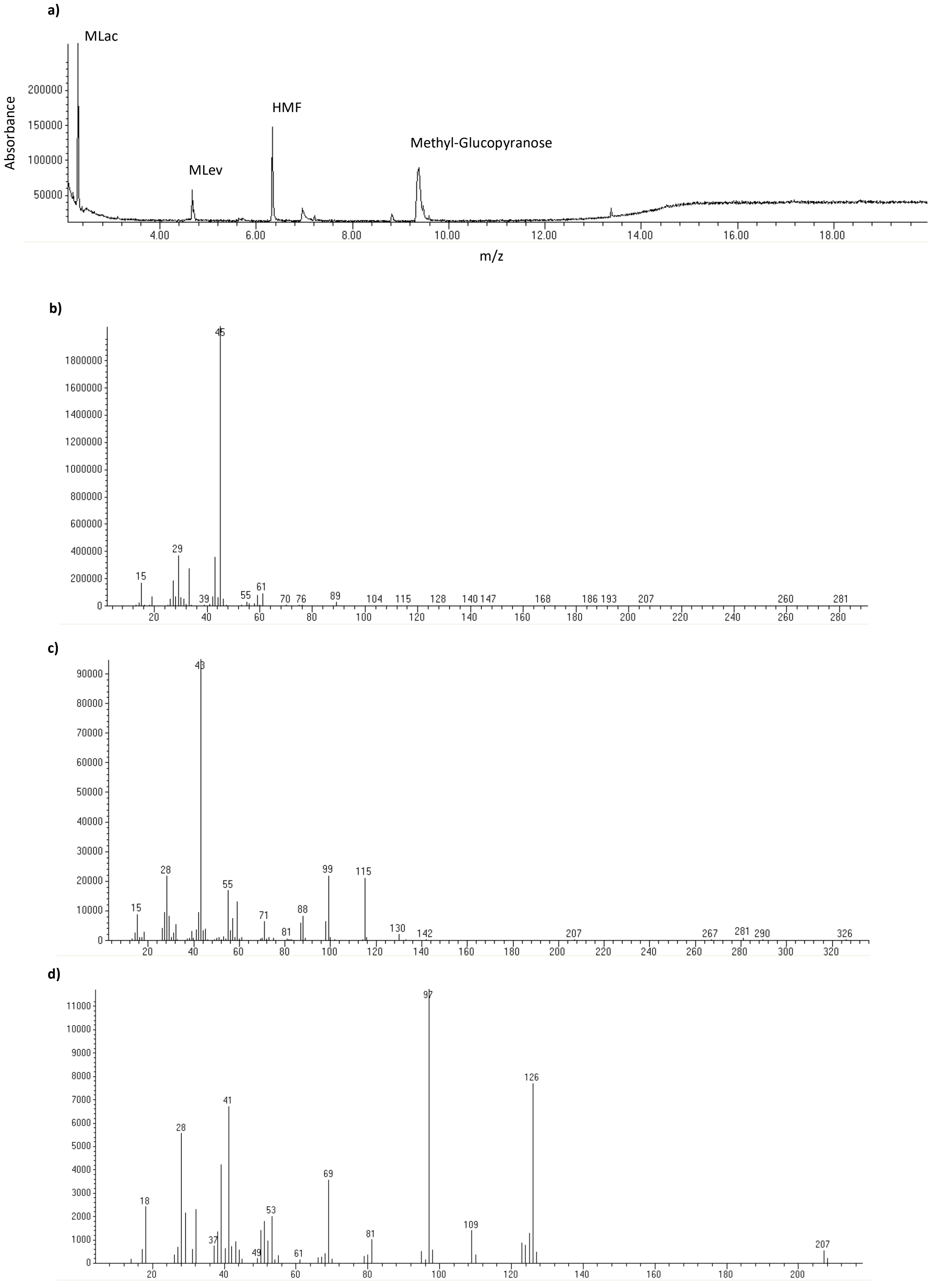

SI 7: *Example GC-MS Chromatogram of the reaction mixture. Reaction Conditions: 100mg glucose, 30mg $SnCl_4 \cdot 6H_2O$ with 2mL MeOH & 1mL H_2O, 15min MW 180^oC a) GC Chromatogram showing product retention times b) MS spectrum of Methyl Lactate c) MS spectrum of Methyl Levulinate d) MS spectrum of hydroxymethylfurfural (HMF)*

5 Metal Chloride Screening Trials for methyl lactate

Table SI 6: *Yields for catalyst candidates screened for methyl lactate production*

Catalyst	Yield (mol%)
$ZrCl_4$	6.7
H_2MoO_4	4.4
$MnCl_2 \cdot 4H_2O$	4.2
$NiCl_2 \cdot 6H_2O$	5.2
$GeCl_2$	4.3
CsCl	4.0
$InCl_3$	5.8
$YbCl_3$	5.1
$AlCl_3 \cdot 6H_2O$	3.5
$SnCl_4 . 5H_2O$	24.8

Reaction conditions: 200mg Glucose, 30mg Catalyst, 3mL MeOH, 160°C for 15 minutes MW

6 Additional Contour plots for Lactic acid, HMF and Methyl levulinate

6.1. Lactic Acid

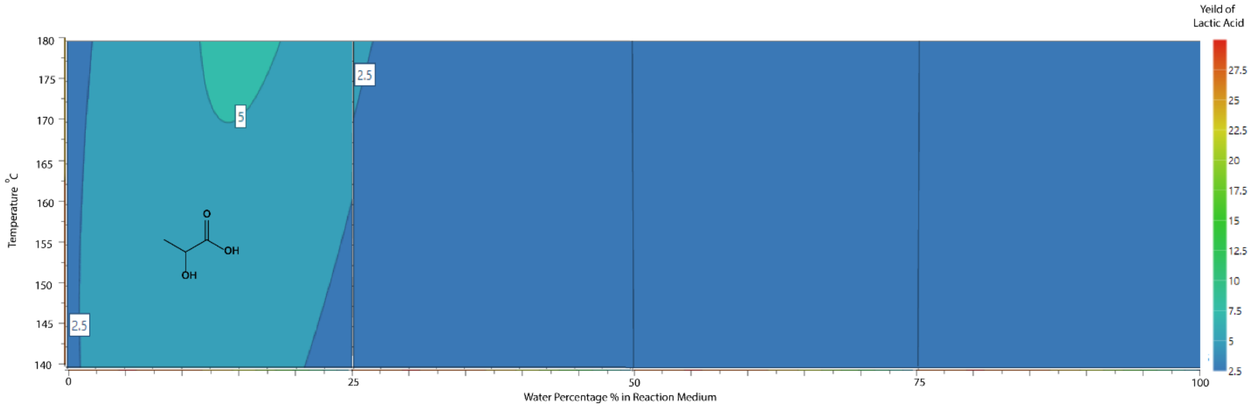

SI 8: *Response contour plot of lactic acid yield (mol%) as a response to water percentage (3mL total volume) and temperature. Reaction conditions: Glucose 0.5mmol,* $SnCl_4 \cdot 5H_2O$ *15mol%, 3mL reaction volume* $MeOH/H_2O$*, temperature 140-180°C, 15min MW*

6.2 HMF

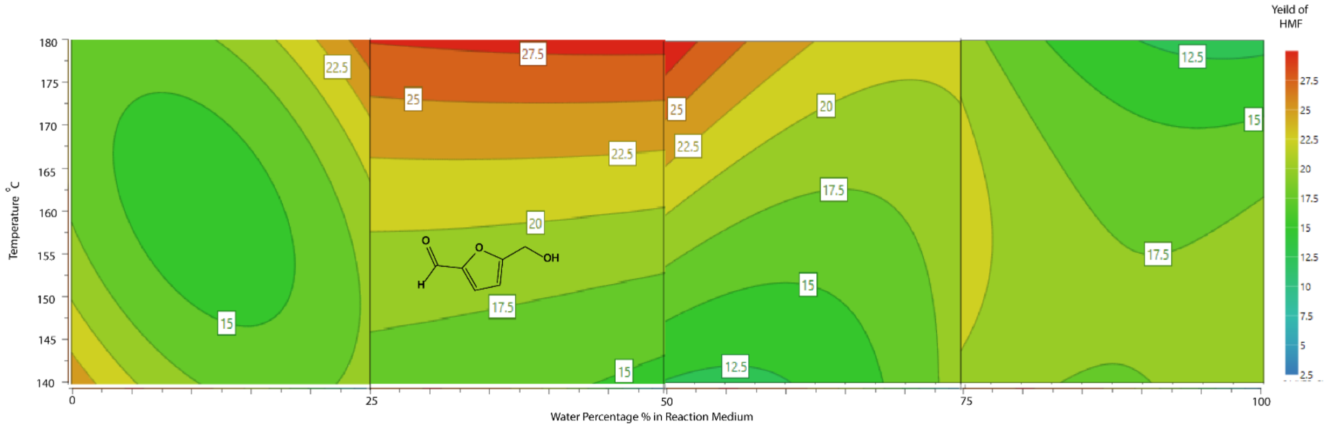

SI 9: *Response contour plot of HMF yield (mol%) as a response to water percentage (3mL total volume) and temperature. Reaction conditions: Glucose 0.5mmol,* $SnCl_4 \cdot 5H_2O$ *15mol%, 3mL reaction volume* $MeOH/H_2O$*, temperature 140-180°C, 15min MW*

6.3 Methyl Levulinate

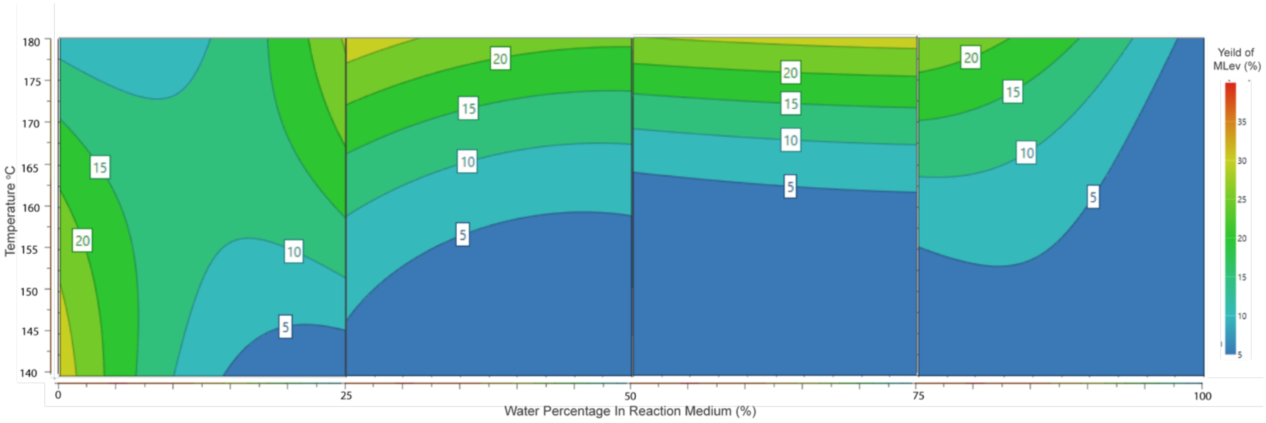

SI 10: *Response contour plot of Methyl Levulinate yield (mol%) as a response to water percentage (3mL total volume) and temperature. Reaction conditions: Glucose 0.5mmol, $SnCl_4{\cdot}5H_2O$ 15mol%, 3mL reaction volume $MeOH/H_2O$, temperature 140-180°C, 15min MW*

7 X-Ray Diffraction Spectrum

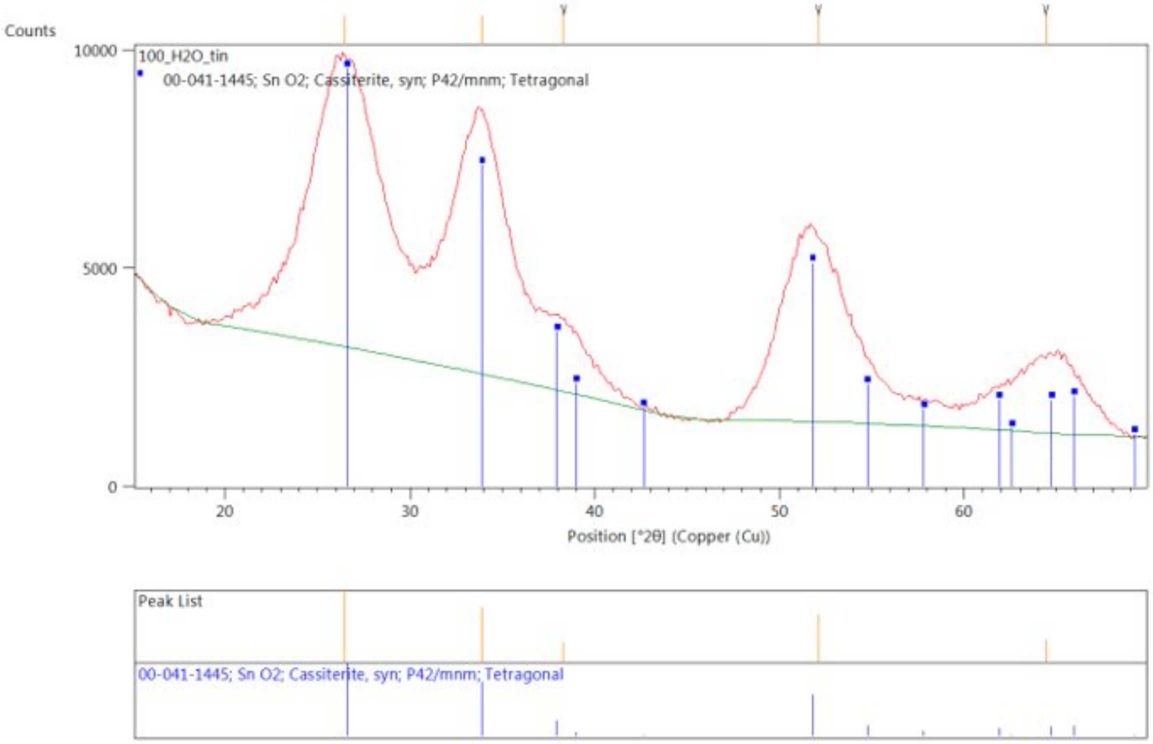

SI 11: *XRD* of $SnCl_45H_2O$ after heating in H_2O at 160°C for 15 mins MW with SnO_2 (00-041-1445) peak signal correlation
